# Random forest method for estimation of brake specific fuel consumption

**DOI:** 10.1038/s41598-023-45026-1

**Published:** 2023-10-18

**Authors:** Qinsheng Yun, Xiangjun Wang, Chen Yao, Haiyan Wang

**Affiliations:** 1 Naval University of Engineering, Wuhan, 430000 China; 2 Shanghai Marine Diesel Engine Research Institute, Shanghai, 200000 China; 3 Shanghai Maritime University, Shanghai, 200000 China

**Keywords:** Electrical and electronic engineering, Mechanical engineering, Engineering

## Abstract

The internal combustion engine is a widely used power equipment in various fields, and its energy utilization is measured using brake specific fuel consumption (BSFC). BSFC map plays a crucial role in the analysis, optimization, and assessment of internal combustion engines. However, due to cost constraints, some values on the BSFC map are estimated using techniques like K-nearest neighbor, inverse distance weighted interpolation, and multi-layer perceptron, which are recognized for their limited accuracy, particularly when dealing with distributed sampled data. To address this, an improved random forest method is proposed for the estimation of BSFC. Polynomial features are employed to increase higher dimensions of features for random forest by nonlinear transformation, and critical parameters are optimized by particle swarm optimization algorithms. The performance of different methods was compared on two datasets to estimate 20%, 30%, and 40% of BSFC data, and the results reveal that the method proposed in this paper outperforms other common methods and is suitable for estimating the BSFC map.

## Introduction

The internal combustion engine finds extensive application in automobiles, ships, agriculture, modern industry, and construction machinery. It operates by converting gas expansion into mechanical energy and is considered the most promising product for energy conservation and emission reduction. To measure its energy efficiency, the brake specific fuel consumption (BSFC) is used. This refers to the fuel consumption of the engine per kilowatt-hour of work and is crucial for improving the engine's economy and thermal efficiency as a heat engine^1,2^. The BSFC map is generated by plotting the fuel consumption against the engine speed and load on the X and Y axes, respectively, over the engine’s operating range. The map serves as an important tool for evaluating engine performance and enhancing its design and efficiency^3,4^.

The BSFC map is a widely used tool in the analysis, optimization, and control of internal combustion engines. It serves multiple purposes, the first of which is to analyze engine performance and predict fuel consumption. This is exemplified by the analysis of a two-circuit bottom cycle system for a diesel engine^5^, where fine-grain fuel consumption is predicted using the BSFC map^6^. The BSFC map can also be used to optimize fuel consumption and reduce engine emissions. For instance, in the automotive industry, the BSFC map is utilized to control diesel engines for minimum fuel consumption^7^ and to obtain the optimal operating mode for the highest economic standard^8^. In addition, the BSFC map is helpful in studying the overall arrangement and system design of internal combustion engines, such as in the modeling and scheduling of fuel-efficient ships^9^.

The BSFC map is an essential tool for internal combustion engine research, and its accurate representation is crucial for further development in this field. Accurate mapping of the BSFC map requires precise measurement of the BSFC, but in practice, some values can only be estimated due to cost and other constraints. Common calculation methods include the K-nearest neighbor (KNN) method, polynomial regression, inverse distance weighted (IDW) method, ordinary kriging (OK) method, and multi-layer perceptron (MLP) method. However, these methods are known to have large errors in estimating uniformly distributed data^10^. This is particularly problematic when drawing high-resolution prediction maps for weather data^11^, where accuracy is essential. Research has shown that these common methods are insufficient for data estimation, especially when the sampled data is intimidatingly distributed, as reported in many fields such as agriculture and mining^12–14^. Recently, machine learning-based methods have become increasingly popular in various fields, including medical imaging and energy^15^. The random forest (RF) method, as an ensemble learning method, uses a decision tree classifier to achieve integrated decision-making^16^. Compared to other machine learning methods, this method has low computation requirements and high precision and is not sensitive to multicollinearity. It also demonstrates good robustness to missing and unbalanced data^10–13^. In this regard, an improved RF method has been introduced in this study to enhance the accuracy of BSFC estimation, marking the first application of this method in estimating the BSFC map. The results show that it outperforms other common methods on two different datasets. Therefore, it is a suitable method for estimating the BSFC map.

## Methods for the estimation of BSFC

The aim of calculating BSFC is to predict the fuel consumption rate of an internal combustion engine under unknown operating conditions by learning the relationship between the fuel consumption rate and the known operating conditions. The relationship between the operating conditions and fuel consumption rate is usually determined through experiments. However, experimental limitations such as cost and conditions result in a limited amount of data. Accurately estimating fuel consumption under different operating conditions is crucial for fuel consumption control, which is a critical task in practical internal combustion engine work. Therefore, predicting fuel consumption under varying operating conditions is a fundamental task for optimizing internal combustion engines.

Consider the operating state of an internal combustion engine is represented by the combined measured state variables such as speed and power, denoted as x. The corresponding fuel consumption rate of the engine, denoted as y, and $D = \{ ({\varvec{x}}_{1} ,y_{1} ),({\varvec{x}}_{2} ,y_{2} ),...,({\varvec{x}}_{N} ,y_{N} )\}$. The task of estimating BSFC involves determining the fuel consumption rate, denoted as y, that corresponds to the unmeasured state variables, denoted as x, based on the dataset D. This problem involves establishing a mapping between the input variable and the output variable, and then using this mapping to predict the output value y for a given input value x. This problem can be classified as either a regression problem or an interpolation problem. There are several methods commonly used to solve this problem, including the KNN method, IDW method, OK method, and MLP method. The introductions are provided for each of these methods below.

### KNN method

The KNN method is a conventional and efficient machine learning technique that operates on a simple concept. It calculates the average values of the points located in close proximity to the estimated points in the known dataset. Due to its speed and simplicity, the KNN method has found its application in various interpolation scenarios, such as cloud edge computing^17^.

### IDW method

The IDW method is a conventional and efficient technique for interpolation. Its fundamental concept involves assigning higher weights to the points in the training set closer to the interpolation points. Let the coordinates of n known points be ($X_{i} ,Y_{i} ,Z_{i}$), and i = 1, 2, 3, …, *n*, then the z value at the point (x, y, z) is given as

1 $$z(x,y) = \left\{ {\begin{array}{*{20}c} {Z_{i} } \\ {\frac{{\sum\nolimits_{i = 1}^{n} {Z_{i} d_{i}^{ - 2} } }}{{\sum\nolimits_{i = 1}^{n} {d_{i}^{ - 2} } }}} \\ \end{array} } \right.{\kern 1pt} {\kern 1pt} {\kern 1pt} {\kern 1pt} {\kern 1pt} {\kern 1pt} {\kern 1pt} {\kern 1pt} {\kern 1pt} {\kern 1pt} \begin{array}{*{20}c} \begin{gathered} x = X_{i} ,y = Y_{i} \hfill \\ \hfill \\ \end{gathered} \\ {{\text{otherwise}}} \\ \end{array}$$

where ${d}_{i}^{-2}$ is the inverse of the Euclidean distance from (x, y) to ($X_{i} ,Y_{i}$) squared. The weight in this method follows a normalization condition, and it is evident that the closer a point is to the interpolation point, the higher the weight assigned to it.

### OK method

The OK method is based on the assumption that the data space has uniform expectations and variance. It uses optimal estimation to obtain the data for unknown points. This geostatistical technique is widely applied in fields such as geographical sciences, environmental sciences, and atmospheric sciences. The OK method has been utilized for deposit Cu concentration^14^ and has been reported to provide high-fidelity uncertainty quantification in composite shell dynamics^18^.

### MLP method

The MLP method employs cascaded neurons that use a sigmoid nonlinear function to map the input to output, enabling the approximation of any nonlinear function. Thus, the neural network can approximate any given multivariable continuous function, including drawing characteristic curves for power machines. This method is highly flexible and possesses a strong nonlinear mapping ability, making it a broadly applicable computational technique. It has found use in numerous applications, such as predicting macroclimate index runoff in atmospheric science^19^ and assessing the sensitivity to flood temperature in geographical research^20^.

## Improved RF method for the estimation of BSFC

### KNN method

RF is a regression method based on trees and has the benefits of strong prediction ability, low overfitting risk, and high interpretability^8,9^. This method is computationally efficient and exhibits superior speed and accuracy^14,15^. It has been widely applied in various fields, including environmental science, agriculture, and engineering. For instance, it has been utilized to classify medical images^21^ and predict indoor radon concentration^22^.

RF is one of the widely used ensemble learning methods. It employs a large number of regression trees for ensemble learning, with random attribute selection during the training process. The regression tree serves as the fundamental learner for RF regression. As with other machine learning techniques, in RF, features and labels, are referred to as X and Y, respectively, while N represents the sample number and D represents the training data set. The representation is as follows: $X\, = \,\{ x_{1} ,x_{2} , \ldots ,x_{N} \} ,Y\, = \,\{ y_{1} ,y_{2} , \ldots ,y_{N} \} ,D\, = \,\{ \left( {x_{1} ,y_{1} } \right), \, \left( {x_{2} ,y_{2} } \right), \ldots ,(x_{N} ,y_{N} )\}$. A regression tree corresponds to a partition of the feature space and labels on the partitioned units. Dividing the feature space into M units $R_{1} , \, R_{2} , \ldots ,\;R_{M}$, each unit *R*_*M*_ with a fixed label *C*_*m*_, the regression tree model can be represented as

2 $$f({\varvec{x}}) = \sum\limits_{m = 1}^{M} {c_{m} I({\varvec{x}} \in R_{m} )}$$

3 $$I({\varvec{x}} \in R_{m} ) = \left\{ \begin{gathered} 0{ (}{\varvec{x}} \notin R_{m} {)} \hfill \\ 1{ (}{\varvec{x}} \in R_{m} {)} \hfill \\ \end{gathered} \right.$$

The square error (E) is used to express the prediction error of the regression tree for the training data in the feature space whose partitioning method has been determined.

4 $$E = \sum\limits_{{{\varvec{x}}_{i} \in R_{m} }} {(y_{i} - f({\varvec{x}}_{i} ))^{2} }$$

This error is used to determine the optimal output value on each unit. In the RF method, the following algorithm is used to generate a regression tree.

- Step 1: Select the *j*-th variable and its value s as the segmentation variable and segmentation point, respectively. The two regions are defined as follows:
  5 $$\begin{gathered} R_{1} (j,s) = \left\{ {\left. {\left. {\varvec{x}} \right|{\varvec{x}}^{(j)} \le s} \right\}} \right. \hfill \\ R_{2} (j,s) = \left\{ {\left. {\left. {\varvec{x}} \right|{\varvec{x}}^{(j)} > s} \right\}} \right. \hfill \\ \end{gathered}$$
- Step 2: Solve the following problem to obtain the optimal *j* and *s* values. These values divide the input space into two regions, *R*_*1*_ and *R*_*2*_.
  6 $$\mathop {min}\limits_{j,s} \left[ {\mathop {min}\limits_{{c_{1} }} \sum\limits_{{{\varvec{x}}_{i} \in R_{1} (j,s)}} {(y_{i} - c_{1} )^{2} + \mathop {min}\limits_{{c_{2} }} \sum\limits_{{{\varvec{x}}_{i} \in R_{2} (j,s)}} {(y_{i} - c_{2} )^{2} } } } \right]$$

It is easy to understand that the optimal value $\hat{c}_{m}$ of $c_{m}$ on a unit $R_{m}$ is the mean of the outputs $y_{i}$ corresponding to all input instances $x_{i}$ in the unit, which can be expressed as

7 $$\widehat{c}_{m} = \frac{1}{K}\sum\limits_{k = 1}^{K} {y_{k} ({\varvec{x}}_{k} \in R_{m} )}$$

- Step 3: Repeat steps 1 and 2 for R1 and R2, respectively, until the termination condition is reached. The termination condition can be that each interval contains one sample, all samples have been used, or the number of units has reached a specified number.

The RF method involves creating a training subset by randomly sampling D and evaluating the error of the remaining samples. Multiple random trees are then generated using the same method for generating random trees, except that instead of using all features, a specified number of features are randomly selected. A total of NT regression trees were generated, denoted as $\{ f_{1} ({\varvec{x}}),f_{2} ({\varvec{x}}), \ldots ,f_{{N_{T} }} ({\varvec{x}})\}$. If the weight is set to $W = \{ w_{1} ,w_{2} , \ldots ,w_{{N_{T} }} \}$ and $w_{1} = w_{2} = \cdots = w_{{N_{T} }} = 1/N{\text{T}}$, the regression prediction result for feature ***x*** is

8 $$f({\varvec{x}}) = \sum\limits_{i = 1}^{{N_{T} }} {w_{i} f_{i} ({\varvec{x}})}$$

It is evident that the diversity in RF integration arises not only from sample disturbances but also from attribute disturbances. This results in a greater variation between individuals, leading to strong adaptability and anti-interference ability toward the data.

### Improved RF method

In the RF algorithm, decision trees are generated directly from the features. In machine learning, adding some nonlinear features of input data can be an effective way to increase the complexity of the model. Therefore, this study introduces polynomial features that can generate higher dimensions of features and terms related to each other. Polynomial features are a method of increasing dimensionality and performing nonlinear transformations in machine learning. It combines and expands the original features, which improves the model's expression ability and fitting effect.

Let the feature vector be ${\varvec{x}} = [x_{1} ,x_{2} , \ldots ,x_{m} ]$, and define the feature of a polynomial of degree 0 as $\phi_{0} ({\varvec{x}}) = 1$. The *d*-th polynomial feature can be represented by the following iterative formula.

9 $$\begin{aligned} \phi_{d} ({\varvec{x}})& = [\begin{array}{*{20}c} {\phi_{d - 1} ({\varvec{x}})} & {x_{1}^{n} } & {x_{1}^{n - 1} x_{2} } & {\begin{array}{*{20}c} \cdots & {x_{2}^{n} } & \cdots & {\begin{array}{*{20}c} {x_{m - 1} x_{m}^{n - 1} } & {x_{m}^{n} } \\ \end{array} } \\ \end{array} } \\ \end{array} ] \hfill \\ \, &\quad= [\begin{array}{*{20}c} {\phi_{d - 1} ({\varvec{x}})} & {\phi_{d} ^{\prime}({\varvec{x}})} \\ \end{array} ] \hfill \\ \end{aligned}$$

$\phi_{d} ^{\prime}({\varvec{x}})$ is the row vector, that contains one or more variables from all possible $x_{1} ,x_{2} , \ldots ,x_{m}$ variables, with a degree of *d* as a monomial expression.

When the RF method is used to estimate BSFC, the *d*-degree polynomial feature $\phi_{d} ({\varvec{x}})$ of feature ***x*** serves as the input feature of the RF regression model. This can incorporate more combinations of original features into the consideration of generating decision trees, enhancing their fitting and expression abilities.

The polynomial feature $\phi_{d} ({\varvec{x}}_{i} )$ of each feature ${\varvec{x}}_{i}$ is used to form a new training set $\Phi_{d} (D) = \{ (\phi_{d} ({\varvec{x}}_{1} ),y_{1} ),(\phi_{d} ({\varvec{x}}_{2} ),y_{2} ), \ldots ,(\phi_{d} ({\varvec{x}}_{N} ),y_{N} )\}$. The RF model *F* is trained with $\Phi_{d} (D)$, and the features with a proportion of *p* in all features are employed when the nodes split.

For a given feature vector ${\varvec{x}}$, and its polynomial feature, denoted by $\phi_{d} ({\varvec{x}})$, the predicted result value $\hat{y} = F(\phi_{d} ({\varvec{x}}))$ is obtained using model *F*. The map from feature vector ${\varvec{x}}$ to $\hat{y}$ is called a polynomial feature RF model $f_{(d,p)} ({\varvec{x}})$ with hyperparameters $(d,p)$.

### Parameter optimization based on particle swarm algorithm

When polynomial features are introduced, the feature dimension for the feature vector ${\varvec{x}} = [x_{1} ,x_{2} , \ldots ,x_{m} ]$ and polynomial feature $\phi_{d} ({\varvec{x}})$ increases from m to $C_{m + d}^{d} = {{(m + d)!} \mathord{\left/ {\vphantom {{(m + d)!} {(d!m!)}}} \right. \kern-0pt} {(d!m!)}}$. However, too many polynomial features can cause slow training due to a large number of feature dimensions and may lead to overfitting, while too few features can result in underfitting. Thus, the degree d of the polynomial feature needs to be selected carefully.

Similarly, in decision tree generation, the parameter p represents the proportion of features considered to the total number of features. Too many features can lead to model complexity, which can be affected by noise and randomness, while too few features may cause under-fitting, making it difficult to capture complex relationships in the data. Therefore, when polynomial features are introduced, p needs to be selected more carefully.

Since both p and d are critical parameters, particle swarm optimization algorithms can be considered to optimize their combination. The object function is as follows.

10 $$L(d,p) = \frac{1}{N}\sum\limits_{i = 1}^{N} {(y_{i} - f_{(d,p)} ({\varvec{x}}_{i} ))^{2} }$$

The optimization process begins with initialization, where the total number of particles and the number of iterations are specified. Each particle is randomly assigned a position ***pi*** = {*pi*, *di*} and a velocity ***vi*** = {*v*_*pi*_, *v*_*di*_}. The objective function of each particle is then calculated to obtain the individual optimal solution of that particle, and the position of the particle with the smallest objective function is considered the global optimal solution.

In each iteration, the following calculations are performed.

For the* i*-th particle, the objective function of its particle is calculated. If the objective function result is less than the objective function at the position ${\varvec{g}}_{i}^{best} = \{ g_{pi}^{best} ,g_{di}^{best} \}$ of the individual optimal solution, update the individual optimal solution to the current position. If the objective function result is less than the objective function at the global optimal solution position ${\varvec{g}}^{best} = \{ g_{p}^{best} ,g_{d}^{best} \}$, update the global optimal solution to the current position. The velocity and position of the particles are updated as

11 $$v_{pi} \leftarrow \omega v_{pi} + c_{1} r_{1} (p_{pi}^{best} - p_{i} ) + c_{2} r_{2} (g_{p}^{best} - p_{i} )$$

12 $$v_{di} \leftarrow \omega v_{di} + c_{1} r_{1} (p_{di}^{best} - d_{i} ) + c_{2} r_{2} (g_{d}^{best} - d_{i} )$$

13 $$p_{i} \leftarrow p_{i} + v_{pi}$$

14 $$d_{i} \leftarrow d_{i} + v_{di}$$

In the above equation, $\omega$ is the inertia weight, generally set to 0.9. *c*_*1*_ and *c*_*2*_ are the acceleration coefficients, generally set to 2.0. *r*_*1*_ and *r*_*2*_ are randomly selected from [0, 1] at each update.

When the maximum number of iterations is reached, ${\varvec{g}}^{best}$ is the optimal parameter of* p* and *d*.

## Experiments and results

### Experimental data

The data sets used in this paper were obtained from references^23,24^, The data sets actual measurements of two gasoline internal combustion engines, including speed, power and fuel consumption rate. The two engines produced a total of 52 and 80 measured data points, respectively. Tables 1 and 2 show the Speed, power and fuel consumption rate of the engines.

**Table 1:** Speed, power and fuel consumption rate of first engines^23^.

Rpm (r/min)					
P(kW)	be (g/kW h)	P(kW)	be (g/kW h)	P(kW)	be (g/kW h)
1200		1400		1600	
11.0	214.0	12.0	219.0	13.0	227.0
15.0	191.0	15.0	201.5	15.0	214.0
20.0	177.0	20.0	183.0	20.0	192.0
25.0	168.5	25.0	172.0	25.0	178.0
30.0	167.5	30.0	165.5	30.0	169.5
36.0	171.0	35.0	162.5	35.0	165.5
		41.0	163.5	40.0	165.0
				47.0	171.0
1800		2000		2200	
15.0	223.5	20.0	207.0	18.0	248.5
20.0	195.5	30.0	182.5	25.0	214.5
30.0	174.5	35.0	175.0	35.0	189.5
35.0	170.5	40.0	171.5	45.0	176.5
40.0	169.0	45.0	172.5	50.0	173.0
45.0	169.0	50.0	176.5	55.0	172.5
50.0	171.0	58.0	187.0	60.0	174.5
53.0	174.0			65.0	180.0
2500		/		/	
20.0	254.5				
30.0	211.0				
40.0	188.0				
50.0	177.0				
55.0	175.0				
60.0	175.5				
65.0	178.5				
70.0	185.0				
20.0	254.5				
30.0	211.0

**Table 2:** Speed, power and fuel consumption rate of second engines^24^.

Rpm (r/min)					
P(kW)	be (g/kW h)	P(kW)	be (g/kW h)	P(kW)	be (g/kW h)
1400		1600		1800	
58.61	222.8	68.54	222.0	76.96	226.0
51.51	220.4	61.27	221.7	69.42	225.3
46.69	232.4	55.00	235.4	61.88	226.4
40.77	228.5	47.60	226.5	54.47	233.9
34.63	227.8	40.83	230.5	46.06	242.1
29.85	232.6	34.04	236.8	39.35	283.3
27.16	248.5	27.53	249.1	31.61	253.9
23.05	245.9	20.76	276.1	24.90	271.4
17.18	272.4	13.99	407.9	16.87	323.5
11.85	329.7	6.65	487.0	8.69	468.6
2000		2200		2400	
89.13	206.5	96.92	234.7	101.68	174.2
79.64	231.1	87.45	259.8	90.60	242.2
69.68	231.1	77.08	235.5	81.10	252.1
60.92	233.0	67.17	237.6	71.12	287.4
51.18	242.0	56.30	242.8	61.14	253.6
42.95	244.9	46.72	292.3	51.64	263.6
33.55	265.0	36.28	277.9	40.74	290.6
23.98	299.8	26.72	308.7	31.34	316.8
2600		2800		/	
102.91	256.9	92.53	257.9		
93.85	253.7	80.77	295.3		
84.48	253.5	71.10	282.4		
71.96	260.0	61.66	288.7		
61.56	303.8	52.34	301.9		
50.86	280.7	42.69	329.7		
41.98	300.6	34.77	357.0		
31.39	346.6	21.29	475.4		
20.77	435.6	15.48	580.3		
9.28	812.9	6.57	1080.1		

**Figure 1:**
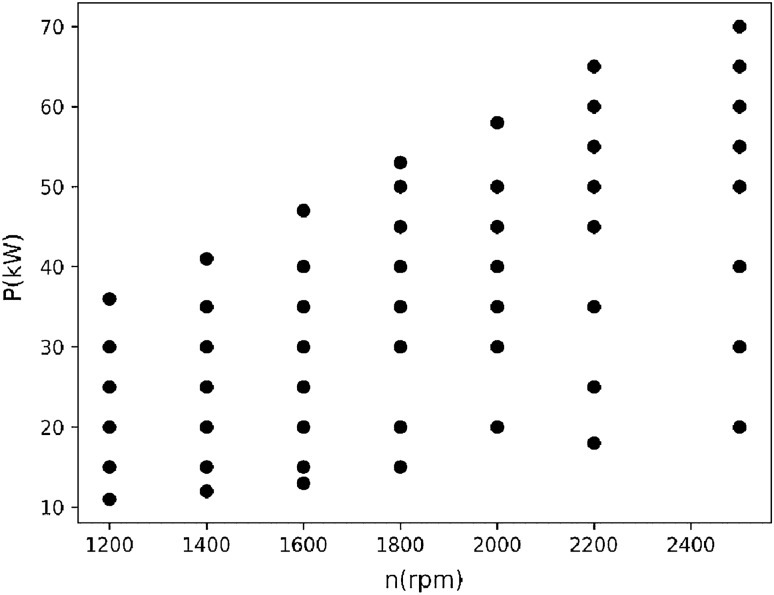
2D distribution of all collected data for the first BSFC.

**Figure 2:**
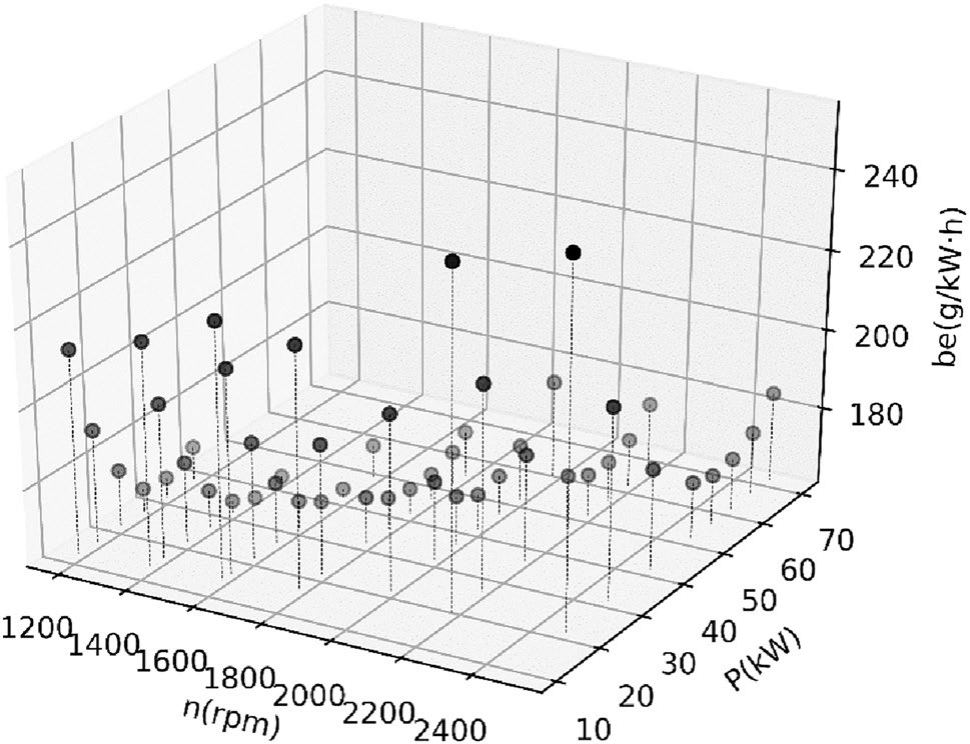
3D view of all collected data for the first BSFC.

Figures 1 and 2 show the distribution of the first fuel engine in the speed-power plane and the distribution of the speed-power-fuel consumption in the three-dimensional space.

**Figure 3:**
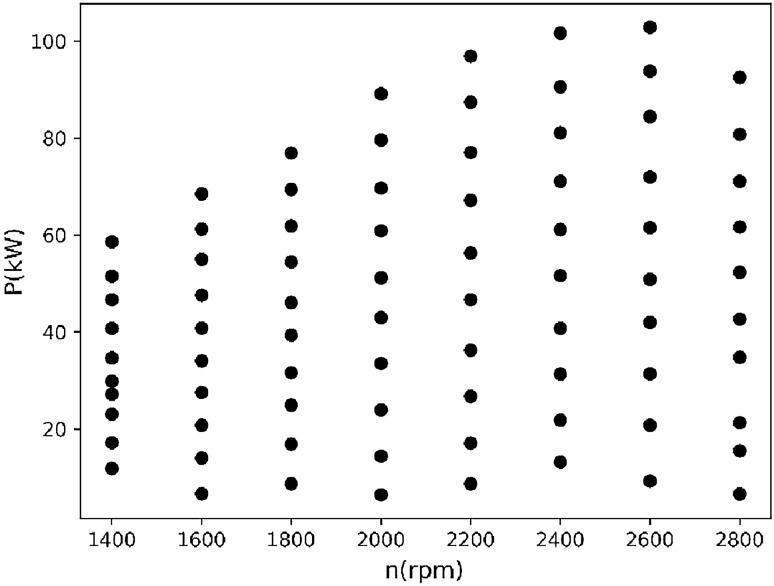
2D distribution of all collected data for the second BSFC.

**Figure 4:**
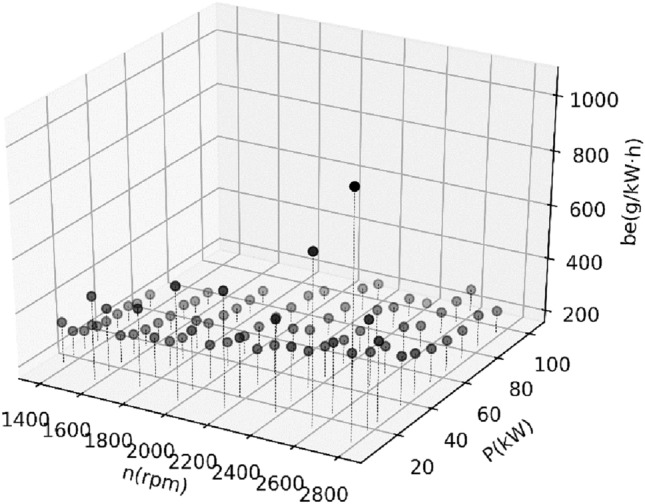
3D view of all collected data for the second BSFC.

Figures 3 and 4 show the distribution of the second fuel engine in the speed-power plane and the distribution of the speed-power-fuel consumption in the three-dimensional space.

### Evaluation index

In this paper, the following indicators are used for evaluation: root mean square error (RMSE), normalized mean square error (NMSE), mean absolute error (MAE), mean absolute percentage error (MAPE), and R-squared (R^2^). Each indicator is calculated as follows.

The RMSE is defined as:

15 $${\text{RMSE}} = \sqrt {{\text{MSE}}}$$

16 $${\text{MSE}} = \frac{1}{n}\sum\limits_{i = 1}^{n} {\left( {y_{i} - \hat{y}_{i} } \right)^{2} }$$

where n is the total number of the data to be estimated, $yi$ is the real value to be estimated, and $yl$ is the estimated value.

To compare the accuracy and degree of variation of different datasets, the NMSE) is proposed to compare the methods on different datasets. The calculation of NMSE is as follows.

17 $${\text{NMSE}} = \frac{{{\text{MSE}}}}{{\frac{1}{n}\sum\limits_{i = 1}^{n} {y_{i}^{2} } }}$$

The MAE is also used to compare estimation errors. The calculation of MAE is

18 $${\text{MAE}} = \frac{1}{n}\sum\limits_{i = 1}^{n} {\left| {y_{i} - \hat{y}_{i} } \right|}$$

To evaluate and compare the accuracy of different algorithms and data sets, the MAPE is utilized in this study. The MAPE is considered more robust than the MAE, as it normalizes the error of each data point and can be used as an evaluation indicator. It is defined as

19 $${\text{MAPE}} = \frac{1}{n}\sum\limits_{i = 1}^{n} {\left| {\frac{{y_{i} - \hat{y}_{i} }}{{y_{i} }}} \right|}$$

R^2^ is also used to evaluate different estimation methods, representing the proportion of estimated data information to original data information. The calculation of R^2^ is as follows.

20 $${\text{R}}^{2} = 1 - \frac{{\sum\limits_{i = 1}^{n} {\left( {y_{i} - \hat{y}_{i} } \right)^{2} } }}{{\sum\limits_{i = 1}^{n} {\left( {y_{i} - \overline{y}} \right)^{2} } }}$$

where $\overline{y }$ represents the average value of all the data to be estimated. The value range of R^2^ is $( - \;\infty ,1]$. The closer R^2^ is to 1, the more accurate the estimation method's results are. On the contrary, the farther R^2^ is from 1, the greater the result error of the estimation method. When R^2^ is less than 1, it indicates that the estimation error of the method is significant, even greater than using the mean as the estimation value.

In this paper, five indicators are used for evaluation, they are RMSE, NMSE, MAE, MAPE, and R^2^. RMSE represents the standard deviation between the estimated value and the true value error, while NMSE represents the percentage of error. MAE represents the average error between the estimated value and the true value, while MAPE represents the percentage of this error. R^2^ expresses the degree of fit between the data and the regression model. NMSE and MAPE can serve as the primary performance indicators, while other indicators can serve as secondary indicators.

### Experimental results

To compare different estimation methods, the known data in this study were randomly divided into two groups at a ratio of 4:1, with 80% of the data being known and the remaining 20% being used for estimation. The data estimation methods compared in this study include KNN, IDW, OK, MLP, RF, and the proposed RF. The performance indicators compared in this study include RMSE, NMSE, MAE, MAPE, and R^2^. To reduce the impact of grouping randomness on statistical results, the experiment was repeated 10 times, using the same ratio for random grouping each time. After each grouping, the known sample dataset and the estimated dataset used for testing have different data. The average of the performance metrics of the 10 experiments is used as the final indicator for performance comparison.

#### Estimating 20% of BSFC data

**Figure 5:**
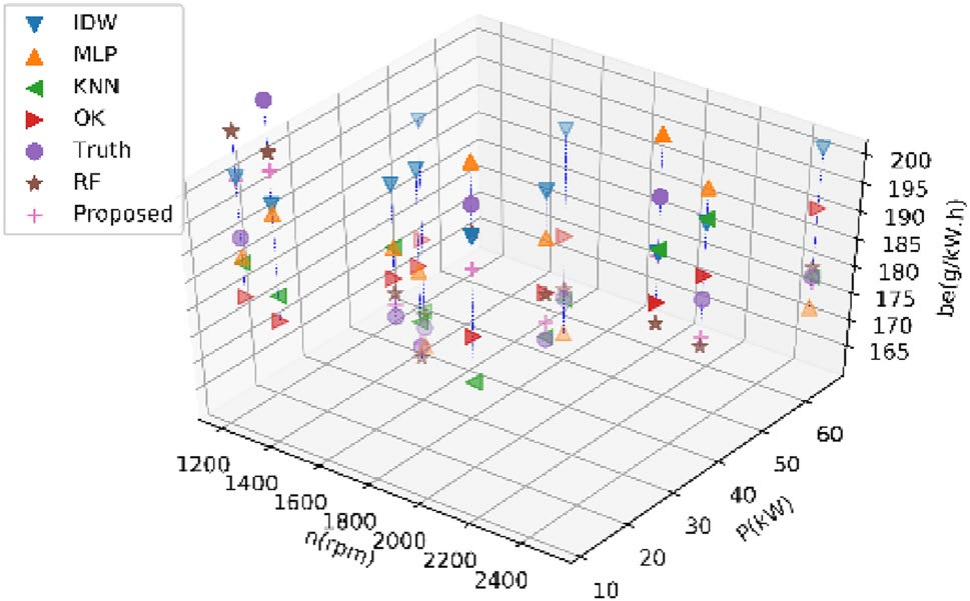
Results of different methods on Dataset 1 for estimating 20% of BSFC data.

**Figure 6:**
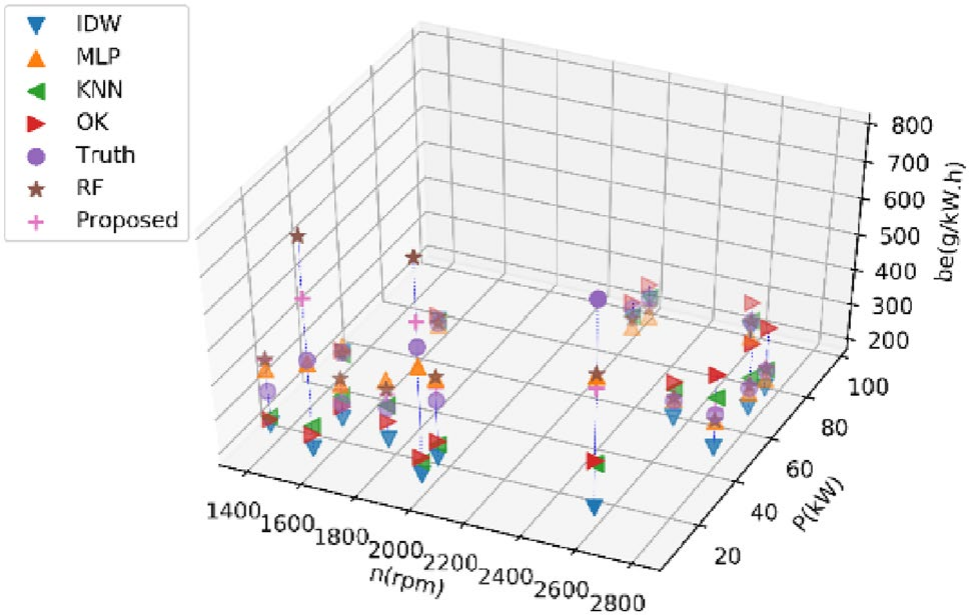
Results of different methods on Dataset 2 for estimating 20% of BSFC data.

Tables 3 and 4 present the performance metrics of various estimation methods on Dataset 1 and Dataset 2 for estimating 20% of BSFC data, respectively. The reported values in these tables are the average results from ten experiments. Figures 5 and 6 display the estimated values of different methods for the BSFC of Datasets 1 and 2, respectively. These figures show the actual estimated result data and real data of a single experiment in the ten experiments.

**Table 3:** Performance comparison of different methods on Dataset 1 for estimating 20% of BSFC data.

Method	RMSE	NMSE	MAE	MAPE	R2
KNN	15.28	0.0079	10.72	0.054	0.29
IDW	26.39	0.0228	10.01	0.105	− 1.66
OK	19.38	0.0114	16.07	0.084	− 0.22
MLP	13.69	0.0092	11.37	0.061	− 0.24
RF	9.81	0.0032	7.00	0.036	0.68
Prposed RF	8.64	0.0025	6.35	0.032	0.75

**Table 4:** Performance comparison of different methods on Dataset 2 for estimating 20% of BSFC data.

Method	RMSE	NMSE	MAE	MAPE	R^2^
KNN	84.19	0.0799	47.54	0.124	0.23
IDW	159.67	0.4993	134.59	0.459	− 5.16
OK	92.46	0.095	68.47	0.209	0.10
MLP	44.09	0.0248	36.00	0.119	0.76
RF	57.68	0.0388	35.12	0.097	0.64
Proposed RF	34.38	0.0143	23.75	0.073	0.87

The results of the experiment conducted on Dataset 1 indicate that the proposed RF method described in this paper outperforms RF method with an RMSE of 0.46 lower, and it outperforms other methods with an RMSE of 5.05 lower. And additionally, the other errors are similar, and the R2 value of RF is closest to 1. These indexes show that the proposed RF has a minimal error and the highest accuracy. Similar results were observed on Dataset 2, the proposed method outperforms other methods with an RMSE of 9.71 lower.

#### Estimating 20% of BSFC data

**Figure 7:**
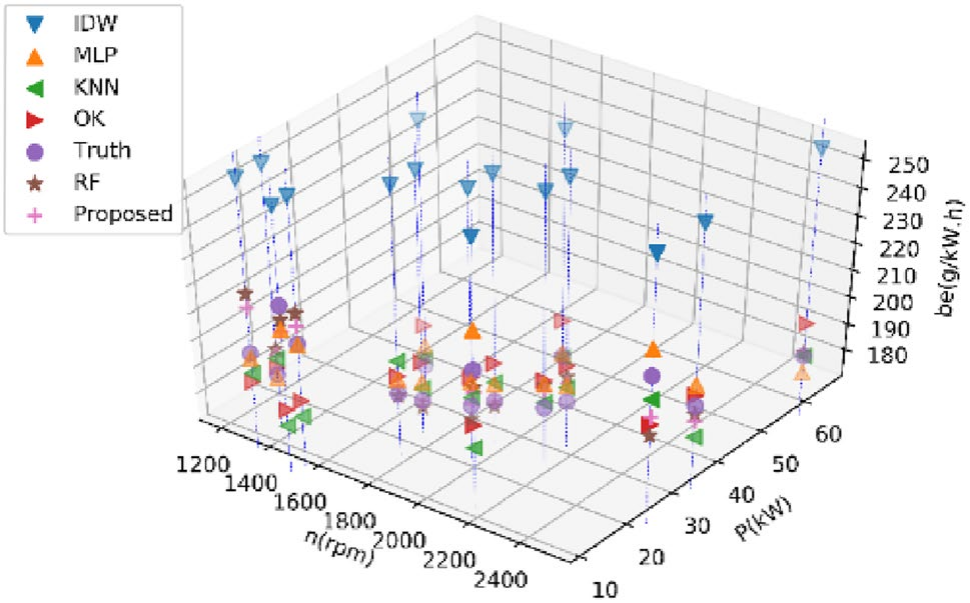
Results of different methods on Dataset 1 for estimating 30% of BSFC data.

**Figure 8:**
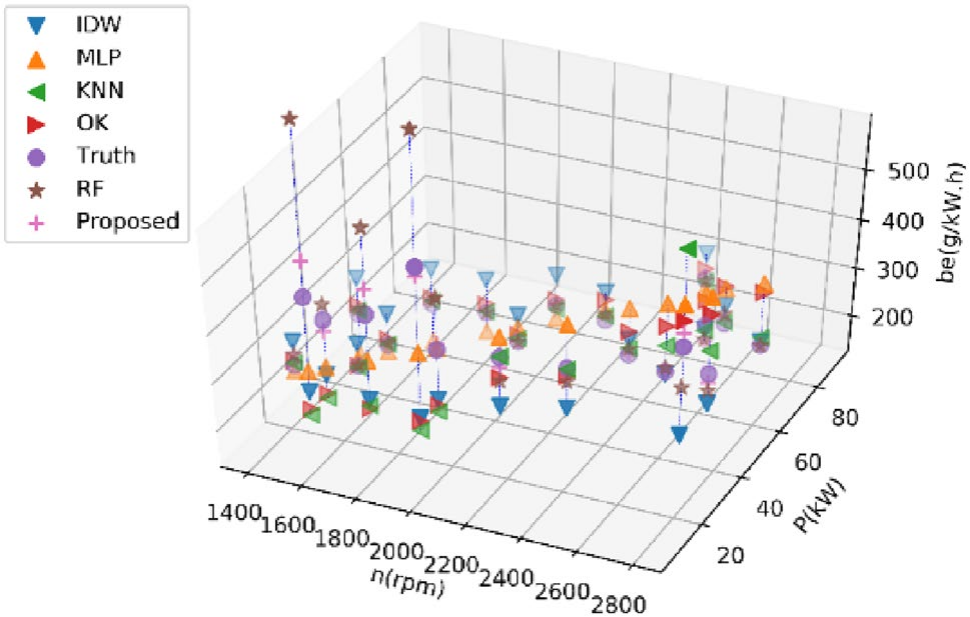
Results of different methods on Dataset 2 for estimating 30% of BSFC data.

Tables 5 and 6 present the average performance metrics of various estimation methods on Dataset 1 and Dataset 2 for estimating 30% of BSFC data after ten experiments, respectively. Figures 7 and 8 display the estimated values of different methods for the BSFC of Datasets 1 and 2 in a single experiment, respectively. The results of the experiment conducted on Dataset 1 indicate that the proposed RF method described in this paper outperforms other methods with an RMSE of 0.66 lower. The proposed method outperforms other methods with an RMSE of 23.84 lower on Dataset 2. All the indexes show that the proposed RF method has a minimal error and the highest accuracy.

**Table 5:** Performance comparison of different methods on Dataset 1 for estimating 30% of BSFC data.

Method	RMSE	NMSE	MAE	MAPE	R^2^
KNN	15.81	0.0076	11.53	0.060	− 0.01
IDW	27.38	0.0302	22.98	0.124	− 2.75
OK	17.97	0.0097	15.42	0.083	− 0.32
MLP	8.58	0.0026	7.00	0.037	0.64
RF	10.34	0.0034	7.89	0.042	0.38
Proposed RF	7.92	0.0021	6.30	0.033	0.64

**Table 6:** Performance comparison of different methods on Dataset 2 for estimating 30% of BSFC data.

Method	RMSE	NMSE	MAE	MAPE	R^2^
KNN	95.76	0.1048	51.53	0.133	0.06
IDW	143.36	0.2790	105.11	0.333	− 2.04
OK	102.06	0.1220	69.59	0.210	− 0.04
MLP	59.88	0.0450	45.53	0.148	0.55
RF	61.51	0.0465	33.97	0.094	0.56
Proposed RF	36.04	0.0178	21.16	0.064	0.86

#### Estimating 40% of BSFC data

**Figure 9:**
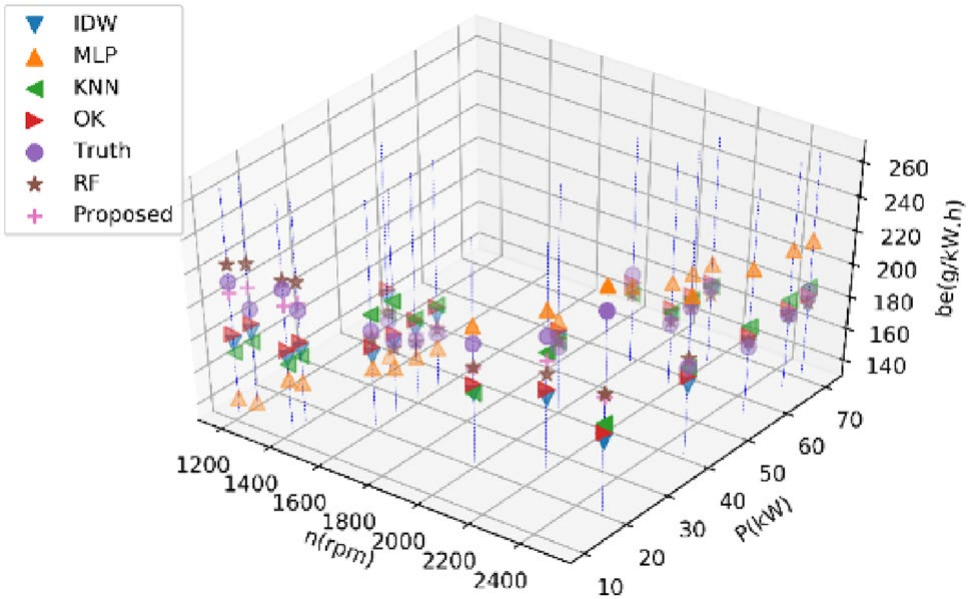
Results of different methods on Dataset 1 for estimating 40% of BSFC data.

**Figure 10:**
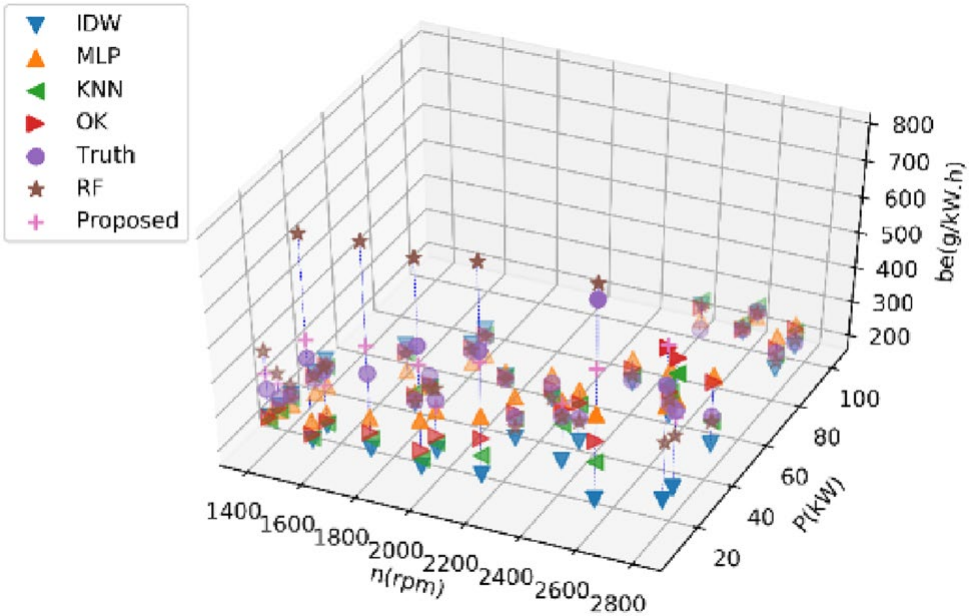
Results of different methods on Dataset 2 for estimating 40% of BSFC data.

Tables 7 and 8 present the average performance metrics of various estimation methods on Dataset 1 and Dataset 2 for estimating 40% of BSFC data after ten experiments, respectively. Figures 9 and 10 display the estimated values of different methods for the BSFC of Datasets 1 and 2 in a single experimen, respectively. The proposed RF method described in this paper outperforms other methods with an RMSE of 1.39 lower on Dataset 1. The proposed method outperforms other methods with an RMSE of 18.25 lower on Dataset 2. All the indexes show that the proposed RF has a minimal error and the highest accuracy.

**Table 7:** Performance comparison of different methods on Dataset 1 for estimating 40% of BSFC data.

Method	RMSE	NMSE	MAE	MAPE	R^2^
KNN	19.95	0.0119	13.76	0.070	− 0.26
IDW	25.97	0.0226	20.89	0.111	− 1.37
OK	20.34	0.0121	15.96	0.083	− 0.31
MLP	17.24	0.0114	14.20	0.076	− 0.02
RF	10.93	0.0037	7.60	0.039	0.63
Proposed RF	9.54	0.0028	6.55	0.033	0.73

**Table 8:** Performance comparison of different methods on Dataset 2 for estimating 40% of BSFC data.

Method	RMSE	NMSE	MAE	MAPE	R^2^
KNN	92.66	0.0952	56.46	0.158	0.08
IDW	159.96	0.3705	127.32	0.429	− 3.23
OK	94.46	0.0988	69.50	0.214	0.04
MLP	59.27	0.0412	48.50	0.159	0.61
RF	72.29	0.0593	39.27	0.105	0.43
Proposed RF	41.02	0.0196	25.61	0.078	0.80

The performance of different methods was compared on two datasets to estimate 20%, 30%, and 40% of BSFC data. All performance indicators indicate that the improved method proposed in this paper is the most accurate.

#### Comparison of NMSE and MAPE

**Figure 11:**
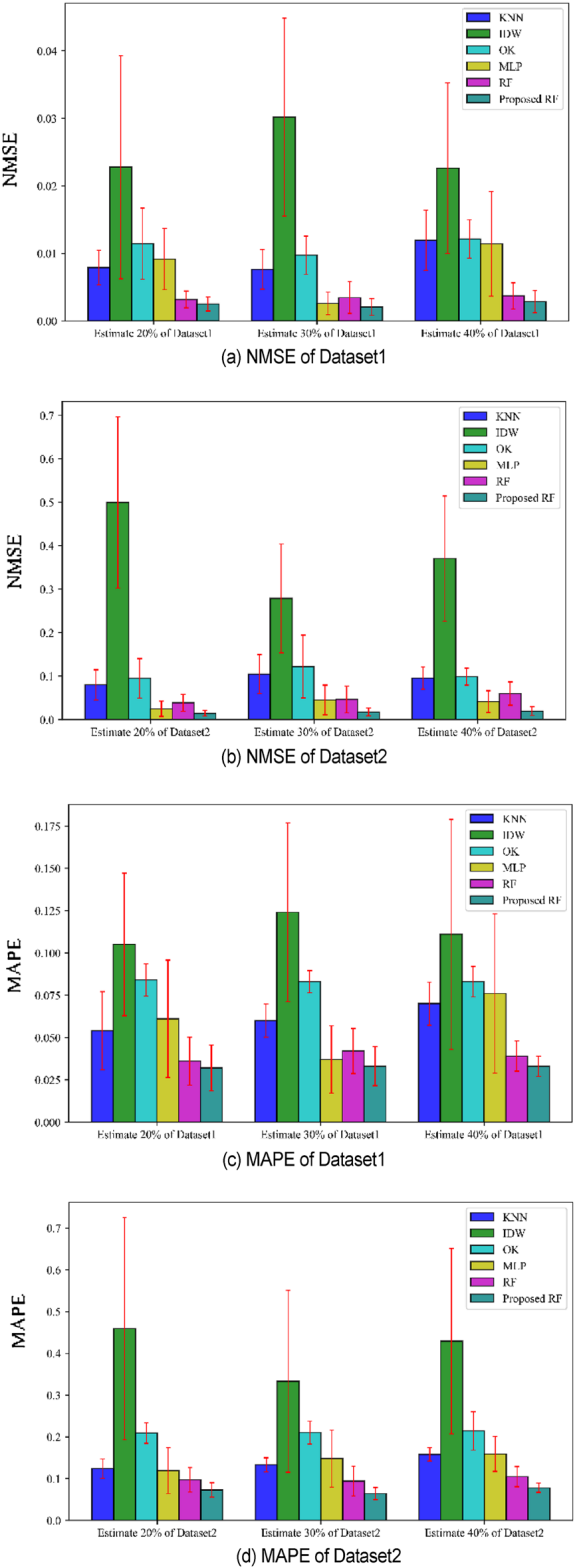
Comparison of NMS and MAPE with standard deviation.

In order to analyze the distribution of performance indicators, the standard deviation of evaluation indicators is calculated. The two most important indicators, NMSE and MAPE, were selected to draw Fig. 11 and added to the paper. In this graph, bar charts with different methods, datasets, and estimated proportions of data were drawn, especially with standard deviations marked in the graph.

From Fig. 11, it can be seen that the improved random forest method proposed in this paper has the minimum average NMSE and MAPE on both dataset 1 and dataset 2. The sample standard deviations of NMSE and MAPE are also shown in the figure, indicating that the proposed method also has the smallest sample standard deviation. From Fig. 11, it can be seen that the improved random forest method proposed in this paper has the minimum average NMSE and MAPE on both dataset 1 and dataset 2. The sample standard deviations of NMSE and MAPE are also shown in the figure, indicating that the proposed method also has the smallest sample standard deviation. This analysis result is consistent with the previous analysis results.

## Conclusions

The random forest method was introduced as an alternative approach for estimating brake-specific fuel consumption and was compared to commonly used calculation methods, such as the K-nearest neighbor method, inverse distance weighted method, ordinary kriging method, and multi-layer perceptron. The experimental results indicated that the proposed RF method outperformed the other methods in accuracy and precision. Therefore, it was concluded that the proposed RF method is more suitable for estimating the BSFC map compared to the other methods.

## Data Availability

All data generated or analyzed during this study are included in this published article.

## List of symbols

*E*: (Effective) work potential
*E*_0_: Exergy
*E*_00_: Energy of a system
K: Kelvin temperature scale
S: Entropy
T: Temperature or Celsius temperature scale
W: Effective work
